# Metformin increases skeletal muscle lactate production in pigs: a microdialysis study

**DOI:** 10.1186/cc9784

**Published:** 2011-03-11

**Authors:** A Protti, P Properzi, S Magnoni, A Santini, T Langer, S Guenzani, P Bertoli, N Stocchetti, L Gattinoni

**Affiliations:** 1 Università degli Studi di Milano, Milan, Italy; 2 Fondazione IRCCS Ca' Granda, Ospedale Maggiore Policlinico, Milan, Italy; 3 Università degli Studi di Milano, Centro Ricerche Chirurgiche Precliniche, Milan, Italy

## Introduction

Lactic acidosis during metformin intoxication is mainly attributed to impaired hepatic lactate clearance [1]. The aim of this present work was to clarify whether metformin at high dose also increases skeletal muscle lactate production.

## Methods

Reverse microdialysis was used in six healthy, sedated and mechanically ventilated pigs, equipped with two skeletal muscle catheters (CMA Microdialysis AB, Sweden). Following a baseline recording, a continuous infusion of saline (control) or metformin diluted in saline (1 mol/l) began. Outflow lactate concentration was measured every 3 hours, up to 12 hours.

## Results

**Figure 1:**
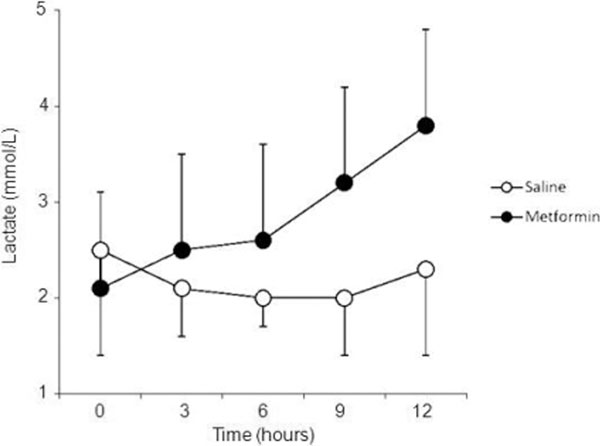
**Muscle lactate level with saline or metformin infused by reverse microdialysis**.

Data are presented as the mean and standard deviation in Figure 1. The interaction between infusion (saline vs. metformin) and time was statistically significant (*P *= 0.02; two-way repeated-measures ANOVA).

## Conclusions

In skeletal muscle, a high dose of metformin increases interstitial lactate levels, a finding consistent with local lactate overproduction.
